# Cross-sectional association of the uric acid-to-HDL cholesterol ratio with diabetic kidney disease in patients with type 2 diabetes

**DOI:** 10.3389/fnut.2026.1887095

**Published:** 2026-07-27

**Authors:** Yongjun Ma, Xinyi Huang, Guangming Chen, Huabin Wang

**Affiliations:** 1 Department of Clinical Laboratory, Affiliated Jinhua Hospital, Zhejiang University School of Medicine, Jinhua, Zhejiang, China; 2 Department of General Practice, Affiliated Jinhua Hospital, Zhejiang University School of Medicine, Jinhua, Zhejiang, China

**Keywords:** diabetic kidney disease, purine-lipid metabolism, renal-metabolic, type 2 diabetes, uric acid-to-HDL cholesterol ratio

## Abstract

**Background:**

Diabetic kidney disease (DKD) is a major complication of type 2 diabetes (T2D) and is closely linked to metabolic dysregulation. The uric acid-to-high-density lipoprotein cholesterol ratio (UHR) integrates urate-related purine metabolism and high-density lipoprotein cholesterol (HDL-C)-related lipid metabolism, two metabolic signals potentially related to kidney injury. This study examined the cross-sectional association between UHR and prevalent DKD in patients with T2D.

**Methods:**

This study included a primary clinical cohort of 912 patients with T2D and an external validation cohort from the National Health and Nutrition Examination Survey (NHANES; *n* = 2,738). Multivariable logistic regression was used to assess the association between UHR and prevalent DKD. Restricted cubic spline (RCS) analyses evaluated exposure-response patterns. Subgroup, interaction, and joint analyses were performed to examine the association across clinical strata.

**Results:**

In the primary cohort, DKD prevalence increased from 20.52% in the lowest UHR quartile to 55.26% in the highest quartile (*P* < 0.001). After full adjustment, each 1-SD increase in UHR was associated with more than 2-fold higher odds of DKD (OR, 2.22; 95% CI, 1.80–2.77; *P* < 0.001). Compared with Q1, the adjusted ORs for Q2, Q3, and Q4 were 1.29, 2.32, and 6.73, respectively. The trend across quartiles was significant (*P* for trend < 0.001). RCS analyses showed positive overall associations between UHR and DKD. The positive association was generally observed across predefined subgroups, with nominal evidence of interaction by body mass index (BMI) and insulin use. In NHANES, 1,067 of 2,738 participants had DKD. External validation showed directionally consistent results. Higher odds of DKD were observed per 1-SD increase in UHR (OR, 1.44; 95% CI, 1.23–1.68; *P* < 0.001) and in Q4 vs. Q1 (OR, 2.61; 95% CI, 1.79–3.79; *P* < 0.001). Survey-weighted RCS showed a positive association without significant non-linearity.

**Conclusion:**

Higher UHR was associated with greater odds of prevalent DKD in the hospital-based T2D cohort, with directionally consistent findings in the NHANES validation cohort.

## 1 Introduction

Diabetic kidney disease (DKD) is a common complication of diabetes and remains a major contributor to chronic kidney disease and kidney failure worldwide (1–3). Recent estimates suggest that the rate of diabetes-related chronic kidney disease continues to rise, in parallel with the increasing prevalence of type 2 diabetes (T2D) (1). In clinical practice, albuminuria and estimated glomerular filtration rate (eGFR) remain the main measures for defining and monitoring kidney involvement in diabetes (4, 5). However, DKD cannot be fully explained by reduced filtration or albuminuria alone. Metabolic dysregulation, oxidative stress, inflammation, endothelial dysfunction, and renal microvascular injury are also involved (6, 7). These mechanisms support the search for routine biochemical indicators associated with the metabolic abnormalities accompanying DKD.

Serum uric acid (UA) and high-density lipoprotein cholesterol (HDL-C) are two conventional laboratory parameters with distinct biological implications. UA, beyond being the end product of purine metabolism, has been associated with oxidative stress, endothelial injury, inflammatory activation, and altered renal hemodynamics (8). These processes are closely related to renal microvascular damage and may contribute to albuminuria and impaired kidney function in diabetes (9). In contrast, HDL-C is generally linked to reverse cholesterol transport, antioxidative activity, anti-inflammatory signaling, and vascular protection (10, 11). However, lipid metabolism in DKD is complex, and diabetes-related changes in HDL quantity and function may weaken these protective properties (12). Therefore, the relative balance between urate-related metabolic stress and HDL-related vascular protection may reflect part of the metabolic milieu associated with DKD.

The UA-to-HDL cholesterol ratio (UHR) integrates these two biological signals into a simple composite index. Because both components are routinely measured, UHR may provide a simple composite index related to pro-oxidative, inflammatory, and lipid-related metabolic imbalance (13). Previous studies have linked UHR with chronic kidney disease, metabolic syndrome, cardiovascular outcomes, and mortality in populations with metabolic disorders (14, 15). Recent population-based work has also suggested a positive association between UHR and DKD (16). Although several studies have reported positive associations between UHR and DKD, the consistency of this association in hospital-based cohorts of patients with T2D remains insufficiently clarified, particularly across different exposure definitions, exposure-response patterns, clinically relevant subgroups, and external validation settings.

Therefore, from a renal-metabolic perspective, we examined the cross-sectional association between UHR and prevalent DKD in a primary clinical cohort of 912 patients with T2D. We assessed UHR as a continuous, standardized, binary, and quartile-based exposure, and further characterized exposure-response patterns using restricted cubic spline (RCS) models. Subgroup, interaction, and joint analyses were performed to evaluate whether the association differed across clinically relevant strata. Finally, we conducted survey-weighted external validation in 2,738 adults with diabetes from the National Health and Nutrition Examination Survey (NHANES) to examine the consistency of the findings in a population-based setting.

## 2 Methods and materials

### 2.1 Study design and data sources

This observational study included a primary hospital-based cross-sectional cohort and an external validation cohort from the NHANES 2011–2018. The primary cohort consisted of patients with T2D treated in the Department of Endocrinology, Affiliated Jinhua Hospital, Zhejiang University School of Medicine, between January 2024 and December 2024. In both cohorts, the main exposure was the UHR, and the outcome was prevalent DKD. The primary cohort was used for the main analyses of the association between UHR and DKD in patients with T2D. The NHANES cohort was used as an independent population-based dataset to examine the external consistency of the findings.

### 2.2 Primary clinical cohort

**Figure 1:**
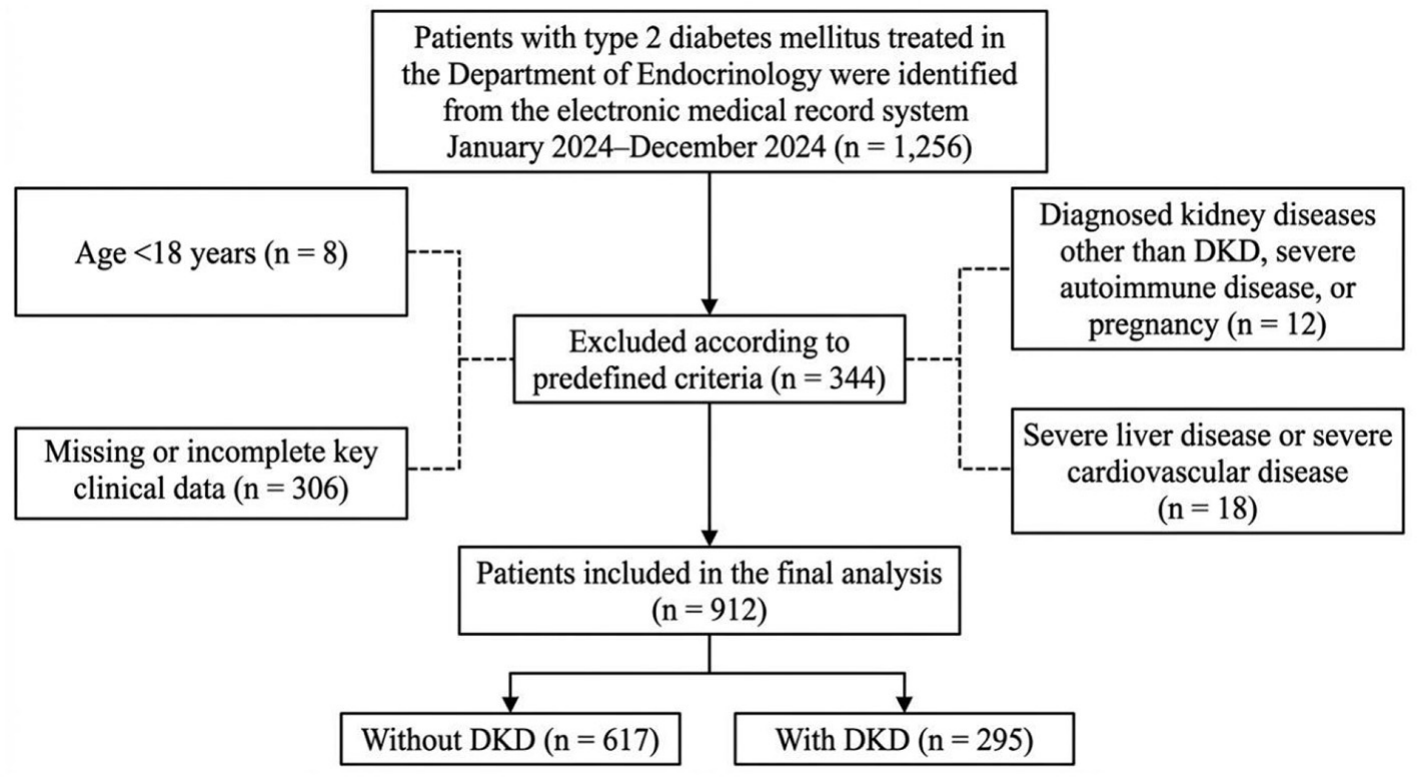
Flowchart of participant selection in the primary cohort. Patients with type 2 diabetes mellitus treated in the Department of Endocrinology between January 2024 and December 2024 were screened from the electronic medical record system. DKD, diabetic kidney disease.

In the primary cohort, patients were screened through the electronic medical record system. The diagnosis of T2D was based on the clinical diagnosis recorded in the medical records. A total of 1,256 patients with T2D were initially identified. Patients were excluded according to predefined criteria, including age younger than 18 years (*n* = 8), diagnosed kidney diseases clearly attributable to causes other than DKD, severe autoimmune disease, or pregnancy (*n* = 12), severe liver disease or severe cardiovascular disease (*n* = 18), and missing or incomplete key clinical data required for defining UHR, DKD status, or covariates, including ACR, serum creatinine, diabetes duration, serum UA, and lipid profiles (*n* = 306). Age ≥18 years was used as the adult eligibility threshold according to the legal and clinical definition of adulthood in China. Exclusion criteria were applied sequentially in the order listed above, and each excluded participant was counted only once according to the first applicable exclusion criterion (Figure 1). Among the included patients, 295 were classified as having DKD and 617 were classified as not having DKD. The participant selection process for the primary cohort was shown in Figure 1. This study was conducted in accordance with the Declaration of Helsinki. The study protocol for the hospital-based cohort was approved by the Ethics Committee of Affiliated Jinhua Hospital, Zhejiang University School of Medicine [Approval No. [Res] 2026-Ethical Review-196]. The requirement for informed consent was waived because of the retrospective nature of the study.

### 2.3 NHANES external validation cohort

For external validation, we used data from the NHANES 2011–2018. NHANES is a population-based survey conducted in 2-year cycles in the United States. A total of 39,156 participants were available in NHANES 2011–2018. After restricting the sample to adults aged ≥20 years, according to the conventional adult definition used in NHANES metabolic and diabetes-related analyses, 22,617 participants remained. Among them, 3,938 adults with diabetes were identified. Diabetes was defined according to self-reported physician diagnosis, use of glucose-lowering medication or insulin, HbA1c ≥6.5%, or fasting plasma glucose ≥7.0 mmol/L, according to data availability. Participants were then excluded if DKD status could not be determined because of missing urinary albumin-to-creatinine ratio (UACR) or eGFR, if serum UA or HDL-C data were unavailable for UHR calculation, or if key covariates were missing. Finally, 2,738 adults with diabetes were included in the NHANES validation cohort. Because NHANES uses a complex, multistage probability sampling design, survey design variables were considered in the validation analyses. The primary sampling unit, strata, and examination weights were specified using SDMVPSU, SDMVSTRA, and MEC examination weights, respectively. Since four 2-year cycles were combined, the 8-year MEC weight was calculated as WTMEC2YR divided by 4. The selection of the NHANES analytic sample was presented in Supplementary Figure S1. NHANES data are publicly available and de-identified. Therefore, the NHANES component of the present study was exempt from additional ethical review by the Ethics Committee of Affiliated Jinhua Hospital, Zhejiang University School of Medicine.

### 2.4 Definitions of exposure, outcome, and covariates

UHR was calculated after expressing both serum uric acid and HDL-C in mmol/L. Serum uric acid values reported in μmol/L were converted to mmol/L before calculation. The formula was: UHR = [serum uric acid (mmol/L)/HDL-C (mmol/L)] × 100. Because it is a ratio of two concentrations expressed in the same unit, UHR was treated as a dimensionless index on a × 100 scale. This calculation was applied consistently in the primary cohort and in the NHANES validation cohort. The outcome was prevalent DKD. In both cohorts, DKD was defined among participants with diabetes as reduced kidney function and/or albuminuria, namely eGFR < 60 mL/min/1.73 m^2^ and/or ACR ≥ 30 mg/g (17). eGFR was calculated from serum creatinine using the Chronic Kidney Disease Epidemiology Collaboration (CKD-EPI) equation. Because repeated measurements were not available, DKD status was determined using single-occasion eGFR and ACR measurements.

In the primary cohort, demographic information, clinical characteristics, medication use, and laboratory measurements were extracted from the electronic medical record system. These variables included age, sex, body mass index (BMI), systolic blood pressure (SBP), diastolic blood pressure (DBP), diabetic duration, hypertension status, insulin use, statin use, sodium-glucose cotransporter-2 inhibitors (SGLT2i) or glucagon-like peptide-1 receptor agonists (GLP-1RA) use, angiotensin-converting enzyme inhibitors (ACEI) or angiotensin II receptor blockers (ARB) use, glycated hemoglobin A1c (HbA1c), fasting plasma glucose (FPG), total cholesterol (TC), triglycerides (TG), low-density lipoprotein cholesterol (LDL-C), HDL-C, serum UA, albumin, total protein, serum creatinine, eGFR, and ACR. BMI was calculated as weight in kilograms divided by height in meters squared. Smoking status, urate-lowering agents, diuretics, diet, alcohol intake, and physical activity were not consistently available in the electronic medical records and therefore could not be reliably included as covariates in the primary cohort analyses.

For the NHANES validation cohort, demographic, questionnaire, examination, and laboratory data were obtained from the corresponding NHANES files. Covariates included age, sex, race/ethnicity, BMI, HbA1c, lipid profiles, blood pressure, diabetic duration, hypertension status, and insulin use, according to data availability. Diabetes duration and insulin use were based on questionnaire information. Hypertension in NHANES was defined using available questionnaire and examination data, including self-reported physician diagnosis, current antihypertensive treatment, or elevated measured blood pressure.

### 2.5 Statistical analysis

Baseline characteristics were summarized as mean ± standard deviation (SD), median with interquartile range, or number with percentage, as appropriate. Characteristics were compared across groups using analysis of variance, *t*-tests, Mann–Whitney *U* tests, Kruskal–Wallis tests, or chi-square tests, as appropriate.

In the primary cohort, multivariable logistic regression was used to estimate odds ratios (ORs) and 95% confidence intervals (CIs) for the association between UHR and prevalent DKD. UHR was analyzed in four forms: per 1-unit increase, per 1-SD increase, median-based low/high groups, and quartiles. Three models were constructed based on clinically relevant factors and previously reported DKD-related covariates (18–20). Model 1 was adjusted for age and sex. Model 2 was further adjusted for HbA1c, BMI, TG, LDL-C, and SBP. Model 3 was additionally adjusted for diabetic duration, hypertension, and insulin use.

RCS analysis was performed to examine the exposure-response association between UHR and DKD, with the median UHR used as the reference value. Because serum creatinine is closely related to renal function and may influence the observed association, an additional sensitivity model was fitted by further adjusting for serum creatinine on the basis of Model 3. To further evaluate potential medication-related confounding, a medication-adjusted sensitivity analysis was performed in the primary cohort by additionally adjusting for SGLT2i/GLP-1RA use and statin use on the basis of Model 3. ACEI/ARB use was not additionally included because all ACEI/ARB users in the primary cohort were hypertensive, and hypertension status had already been included in the multivariable models. This model was used only as a conservative sensitivity analysis, not as the primary model. Subgroup analyses were performed according to sex, age (< 60 or ≥60 years), BMI (< 24 or ≥24 kg/m^2^), hypertension status, HbA1c (< 7% or ≥7%), insulin use, and diabetic duration (< 10 or ≥10 years). Interaction terms between UHR and subgroup variables were added to the fully adjusted model to test for heterogeneity. Joint analyses were further conducted for UHR with BMI and UHR with insulin use. In the UHR–BMI joint analysis, the model was adjusted for age, sex, HbA1c, TG, LDL-C, SBP, diabetic duration, hypertension, and insulin use. In the UHR–insulin use joint analysis, insulin use was not included as an adjustment variable because it was part of the joint grouping. Multicollinearity in the fully adjusted model was assessed using variance inflation factors and Spearman correlation coefficients.

Additional analyses were conducted according to DKD components, including ACR ≥30 mg/g, eGFR < 60 mL/min/1.73 m^2^, and the coexistence of both abnormalities. The main analysis was also repeated after excluding participants with eGFR < 60 mL/min/1.73 m^2^. These analyses used the same covariate adjustment strategy as the fully adjusted model, and RCS analyses were performed as supportive exposure-response analyses.

For the NHANES validation cohort, all analyses accounted for the complex survey design using SDMVPSU, SDMVSTRA, and 8-year MEC examination weights. The 8-year weight was calculated as WTMEC2YR divided by 4. Survey-weighted logistic regression was used to examine the association between UHR and DKD. Weighted cutoffs were used to define UHR median groups and quartiles. Model A was adjusted for age, sex, and race/ethnicity. Model B was further adjusted for BMI, SBP, and HbA1c. Model C was additionally adjusted for diabetic duration, hypertension, and insulin use. These models were generally consistent with the primary cohort models, while race/ethnicity was included for NHANES. TG and LDL-C were not included in the NHANES models because of substantial missing data. Survey-weighted RCS and subgroup analyses were also performed under the fully adjusted Model C. All analyses were performed using R version 4.3.2 (R Foundation for Statistical Computing, Vienna, Austria). A two-sided *P*-value < 0.05 was considered statistically significant.

## 3 Results

### 3.1 Baseline characteristics of the primary cohort according to UHR quartiles

Baseline characteristics according to UHR quartiles are shown in Table 1. Among the 912 participants, 229, 227, 228, and 228 were classified into UHR quartiles Q1–Q4, respectively. Age, SBP, albumin, total protein, and insulin use did not differ significantly across quartiles. Higher UHR levels were associated with a lower proportion of female participants, higher BMI and DBP, and higher prevalences of hypertension and ACEI/ARB use. Significant differences were also observed in HbA1c, FPG, LDL-C, HDL-C, TG, and UA. Renal markers showed a more adverse profile with increasing UHR, as reflected by higher sCr and ACR levels and lower eGFR (all *P* < 0.001). DKD prevalence increased from 20.52% in Q1 to 55.26% in Q4 (*P* < 0.001).

**Table 1:** Baseline characteristics of the study participants according to UHR quartiles.

Variable	UHR Q1 (*n* = 229)	UHR Q2 (*n* = 227)	UHR Q3 (*n* = 228)	UHR Q4 (*n* = 228)	*P*-value
Age (years)	60.34 (12.38)	59.56 (11.65)	60.45 (12.79)	59.13 (14.26)	0.642
Female, *n* (%)	157 (68.56)	103 (45.37)	69 (30.26)	47 (20.61)	< 0.001
BMI (kg/m^2^)	23.35 (3.16)	24.58 (3.97)	25.43 (3.23)	26.17 (4.21)	< 0.001
SBP (mmHg)	136.78 (19.60)	136.99 (17.07)	138.73 (18.67)	140.09 (20.51)	0.205
DBP (mmHg)	76.94 (11.41)	78.14 (11.93)	79.46 (10.61)	80.08 (13.22)	0.023
HbA1c (%)	8.09 (2.28)	8.18 (2.24)	7.51 (1.91)	7.75 (2.04)	0.002
FPG (mmol/L)	7.95 (3.02)	8.06 (3.00)	7.53 (2.51)	7.38 (2.61)	0.027
LDL-C (mmol/L)	3.05 (2.47, 3.71)	2.96 (2.23, 3.48)	2.86 (2.25, 3.39)	2.68 (2.15, 3.24)	< 0.001
HDL-C (mmol/L)	1.39 (1.24, 1.56)	1.13 (1.02, 1.26)	1.04 (0.93, 1.16)	0.88 (0.78, 0.99)	< 0.001
TG (mmol/L)	1.14 (0.83, 1.65)	1.37 (1.03, 1.82)	1.57 (1.08, 2.20)	1.74 (1.22, 2.32)	< 0.001
UA (μmol/L)	233 (203, 263)	280 (255, 313)	335 (302, 371)	410 (365, 471)	< 0.001
UHR	17.39 (14.51, 19.12)	24.74 (23.11, 26.04)	32.24 (29.52, 34.51)	44.41 (39.74, 51.90)	< 0.001
Albumin (g/L)	39.57 (4.00)	39.83 (3.96)	40.05 (4.49)	39.22 (5.01)	0.219
Total protein (g/L)	64.79 (6.10)	64.88 (5.09)	65.49 (5.67)	64.95 (6.17)	0.572
sCr (μmol/L)	65.40 (14.83)	71.61 (19.72)	80.25 (25.91)	103.05 (77.39)	< 0.001
eGFR (mL/min/1.73 m^2^)	79.36 (10.41)	79.04 (10.69)	76.55 (11.61)	72.49 (15.64)	< 0.001
ACR (mg/g)	9.89 (6.16, 20.43)	10.04 (4.69, 23.68)	12.21 (5.42, 46.29)	34.08 (8.04, 152.54)	< 0.001
Hypertension, n (%)	102 (44.54)	112 (49.34)	135 (59.21)	152 (66.67)	< 0.001
ACEI/ARB use, *n* (%)	35 (15.28)	53 (23.35)	58 (25.44)	76 (33.33)	< 0.001
Insulin use, *n* (%)	90 (39.30)	69 (30.40)	77 (33.77)	87 (38.16)	0.17
Diabetic duration, years	8.00 (2.00, 13.00)	8.00 (2.00, 13.00)	8.00 (3.00, 13.00)	8.00 (2.00, 15.00)	0.854
SGLT2i/GLP-1RA use, *n* (%)	27 (11.84)	28 (12.28)	21 (9.21)	21 (9.21)	0.578
Statin use, *n* (%)	36 (15.79)	44 (19.30)	32 (14.04)	41 (17.98)	0.447
DKD prevalence, *n* (%)	47 (20.52)	51 (22.47)	71 (31.14)	126 (55.26)	< 0.001

BMI, body mass index; SBP, systolic blood pressure; DBP, diastolic blood pressure; HbA1c, glycated hemoglobin A1c; FPG, fasting plasma glucose; LDL-C, low-density lipoprotein cholesterol; HDL-C, high-density lipoprotein cholesterol; TG, triglycerides; UA, uric acid; UHR, uric acid to high-density lipoprotein cholesterol ratio; sCr, serum creatinine; eGFR, estimated glomerular filtration rate; ACR, albumin-to-creatinine ratio; DKD, diabetic kidney disease; SGLT2i: sodium-glucose cotransporter-2 inhibitors; GLP-1RA: glucagon-like peptide-1 receptor agonists.

### 3.2 Multivariable association between UHR and prevalent DKD in the primary cohort

As shown in Table 2, higher UHR was consistently associated with prevalent DKD across all logistic regression models. For UHR as a continuous variable, each 1-unit increase was associated with higher odds of DKD in Models 1–3, with ORs of 1.06 (95% CI, 1.05–1.08), 1.06 (95% CI, 1.05–1.08), and 1.06 (95% CI, 1.04–1.08), respectively (all *P* < 0.001). Similar associations were observed for each 1-SD increase in UHR, with ORs of 2.27 (95% CI, 1.89–2.75), 2.34 (95% CI, 1.91–2.89), and 2.22 (95% CI, 1.80–2.77) in Models 1–3, respectively (all *P* < 0.001). In categorical analyses, the high-UHR group had higher odds of DKD than the low-UHR group across all models, with ORs ranging from 3.19 to 3.50; in Model 3, the OR was 3.29 (95% CI, 2.28–4.80; *P* < 0.001). In quartile analyses, Q2 was not significantly associated with DKD, whereas Q3 and Q4 showed significant associations. In Model 3, the ORs were 1.29 (95% CI, 0.77–2.16; *P* = 0.33), 2.32 (95% CI, 1.38–3.93; *P* = 0.002), and 6.73 (95% CI, 3.94–11.68; *P* < 0.001) for Q2, Q3, and Q4, respectively, with significant trends across quartiles in all models (all *P* for trend < 0.001).

**Table 2:** Association between UHR and the presence of DKD by logistic regression analyses.

Variables	Model 1 OR (95% CI)	*P*-value	Model 2 OR (95% CI)	*P*-value	Model 3 OR (95% CI)	*P*-value
UHR (per 1 unit)	1.06 (1.05, 1.08)	< 0.001	1.06 (1.05, 1.08)	< 0.001	1.06 (1.04, 1.08)	< 0.001
UHR (per 1-SD)	2.27 (1.89, 2.75)	< 0.001	2.34 (1.91, 2.89)	< 0.001	2.22 (1.80, 2.77)	< 0.001
Median-split						
Low UHR	Reference		Reference		Reference	
High UHR	3.19 (2.33, 4.40)	< 0.001	3.50 (2.46, 5.01)	< 0.001	3.29 (2.28, 4.80)	< 0.001
UHR quartiles						
Q1	Reference		Reference		Reference	
Q2	1.27 (0.80, 2.02)	0.306	1.27 (0.78, 2.08)	0.337	1.29 (0.77, 2.16)	0.33
Q3	2.10 (1.33, 3.33)	0.001	2.34 (1.43, 3.88)	< 0.001	2.32 (1.38, 3.93)	0.002
Q4	6.61 (4.18, 10.62)	< 0.001	7.33 (4.40, 12.41)	< 0.001	6.73 (3.94, 11.68)	< 0.001
*P* for trend		< 0.001		< 0.001		< 0.001

Model 1 was adjusted for age and sex. Model 2 was adjusted for age, sex, HbA1c, BMI, TG, LDL-C, and SBP. Model 3 was further adjusted for diabetic duration, hypertension, and insulin use based on Model 2. UHR, uric acid to high-density lipoprotein cholesterol ratio; DKD, diabetic kidney disease; OR, odds ratio.

In the fully adjusted Model 3, all variance inflation factors were low, ranging from approximately 1.1–1.7 (Supplementary Figure S2A). Pairwise Spearman correlations among model variables were weak to moderate, with a maximum absolute coefficient of 0.41 (Supplementary Figure S2B). These findings did not indicate problematic multicollinearity. Model 3 included 11 parameters for 295 DKD events, corresponding to 26.8 events per variable.

Further adjustment for SGLT2i/GLP-1RA use and statin use did not materially change the association between UHR and prevalent DKD (Supplementary Table S1). In this medication-adjusted sensitivity model, each 1-SD increase in UHR remained associated with higher odds of DKD (OR, 2.21; 95% CI, 1.80–2.75; *P* < 0.001).

### 3.3 Analyses according to DKD phenotypes

Additional analyses according to DKD components were shown in Supplementary Table S2. In the fully adjusted model, higher UHR was associated with ACR ≥30 mg/g (OR per 1-SD increase, 2.05; 95% CI, 1.67–2.53; *P* < 0.001), eGFR < 60 mL/min/1.73 m^2^ (OR, 2.39; 95% CI, 1.81–3.25; *P* < 0.001), and the coexistence of ACR ≥30 mg/g and eGFR < 60 mL/min/1.73 m^2^ (OR, 2.33; 95% CI, 1.75–3.19; *P* < 0.001). When UHR was analyzed by quartiles, participants in the highest quartile had higher odds of these outcomes than those in the lowest quartile. After excluding participants with eGFR < 60 mL/min/1.73 m^2^, the association between UHR and ACR ≥30 mg/g remained significant (OR per 1-SD increase, 1.93; 95% CI, 1.55–2.42; *P* < 0.001; Q4 vs. Q1: OR, 4.39; 95% CI, 2.51–7.80; *P* < 0.001). RCS analyses showed significant overall associations across all four analyses (all *P*-overall < 0.001; Supplementary Figure S3).

### 3.4 Exposure-response pattern between UHR and prevalent DKD assessed by RCS

**Figure 2:**
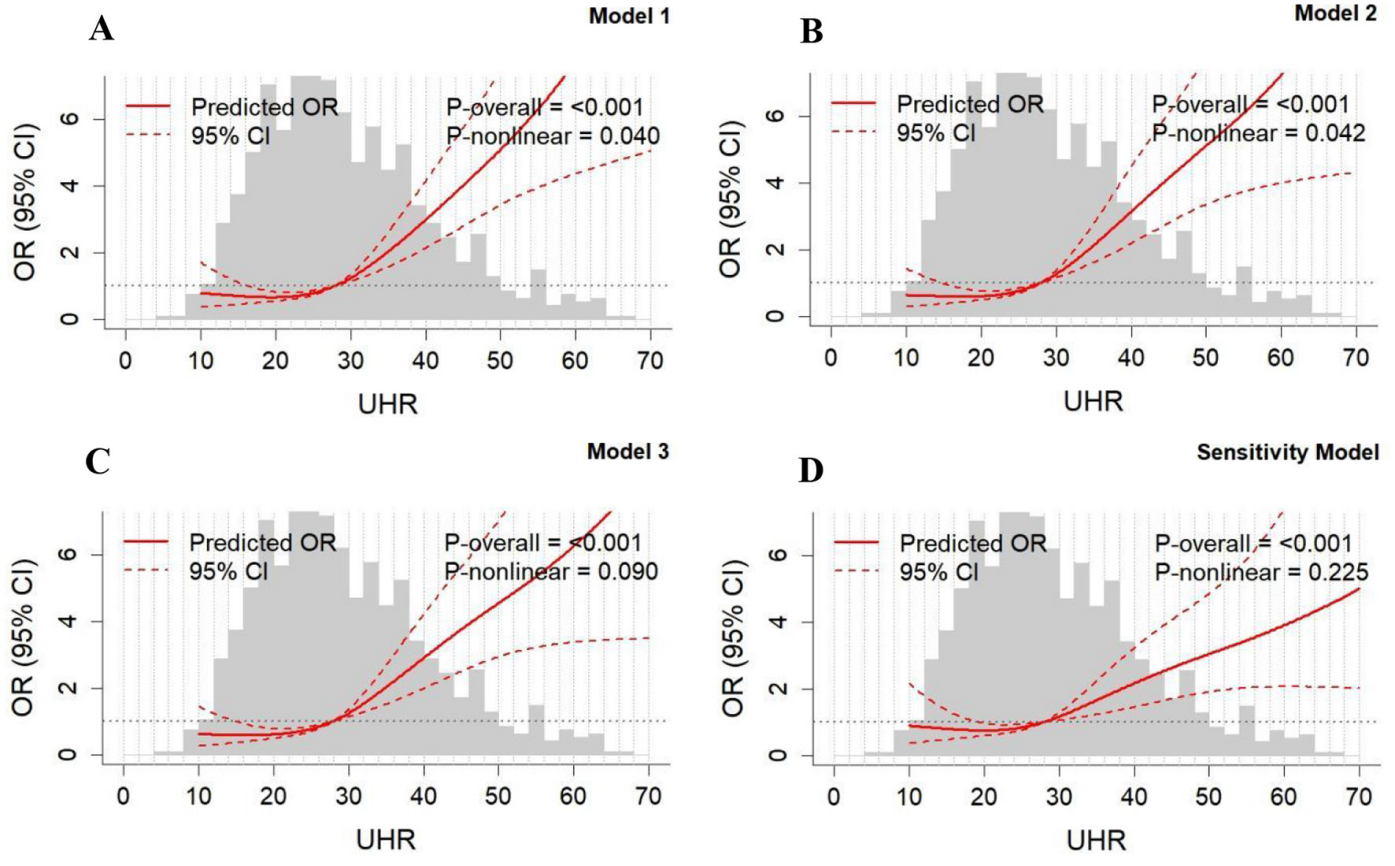
Restricted cubic spline curves for the association between UHR and DKD under different adjustment models. **(A)** Model 1: adjusted for age and sex. **(B)** Model 2: adjusted for age, sex, HbA1c, BMI, TG, LDL-C, and SBP. **(C)** Model 3: further adjusted for diabetic duration, hypertension, and insulin use based on Model 2. **(D)** Sensitivity model: further adjusted for serum creatinine based on Model 3. Red solid lines indicate predicted ORs, red dashed lines indicate 95% CIs, and gray histograms indicate the distribution of UHR.

Figure 2 showed a positive overall association between UHR and DKD across all adjustment models, with significant overall associations throughout (all *P*-overall < 0.001). Evidence of non-linearity was observed in Model 1 (*P*-non-linear = 0.040) and Model 2 (*P*-non-linear = 0.042), but this was attenuated and no longer statistically significant after further adjustment in Model 3 (*P*-non-linear = 0.090) and in the sensitivity model additionally adjusted for serum creatinine (*P*-non-linear = 0.225).

### 3.5 Subgroup, interaction, and joint association analyses in the primary cohort

**Figure 3:**
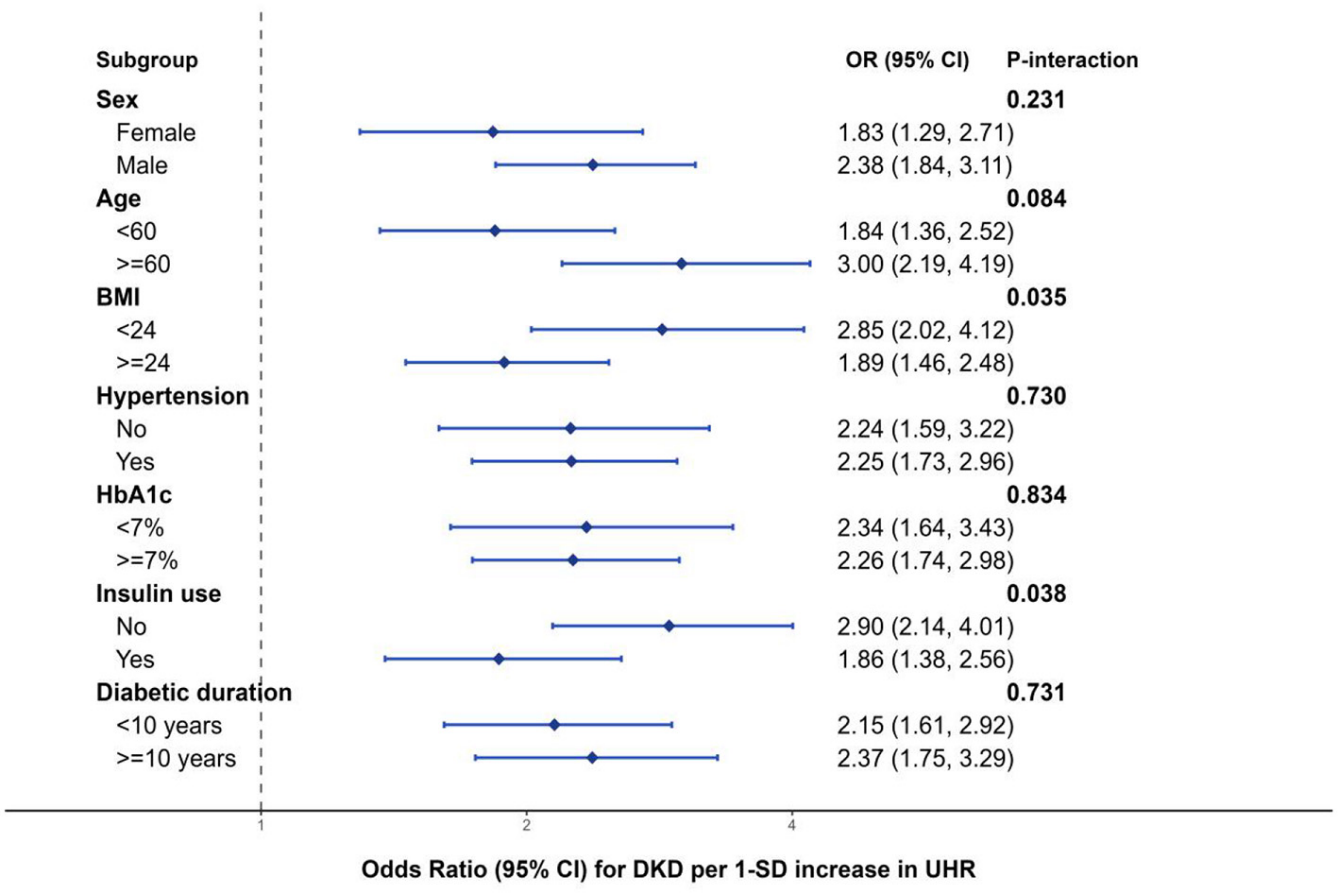
Subgroup analyses of the association between UHR and DKD. Forest plot showing ORs and 95% CIs for DKD per 1-SD increase in UHR across predefined subgroups. Estimates were based on the fully adjusted model (adjusted for age, sex, HbA1c, BMI, TG, LDL-C, SBP, diabetic duration, hypertension, and insulin use), with P values for interaction shown for each subgroup. UHR, uric acid to high-density lipoprotein cholesterol ratio; HbA1c, glycated hemoglobin A1c; BMI, body mass index; TG, triglycerides; LDL-C, low-density lipoprotein cholesterol; SBP, systolic blood pressure.

Subgroup and interaction analyses for each 1-SD increase in UHR are shown in Table 3 and Figure 3. In the fully adjusted model, UHR was significantly associated with higher odds of DKD across all predefined subgroups. No significant interaction was observed for sex, age, hypertension, HbA1c, or diabetic duration, although the interaction by age showed a borderline pattern (*P* for interaction = 0.084). Exploratory subgroup analyses suggested possible heterogeneity by BMI (*P* for interaction = 0.035) and insulin use (*P* for interaction = 0.038), with nominally significant interaction tests. The association was stronger in participants with BMI < 24 kg/m^2^ than in those with BMI ≥24 kg/m^2^ [OR 2.85 (95% CI, 2.02–4.12) vs. 1.89 (95% CI, 1.46–2.48)] and in non-insulin users than in insulin users [OR 2.90 (95% CI, 2.14–4.01) vs. 1.86 (95% CI, 1.38–2.56)].

**Table 3:** Subgroup and interaction analyses of the association between UHR and DKD.

Subgroup	*N*	Events	OR (95%CI)	*P*-value	*P* for interaction
Sex					0.231
Female	376	124	1.83 (1.29, 2.71)	0.001	
Male	536	171	2.38 (1.84, 3.11)	< 0.001	
Age					0.084
< 60	451	116	1.84 (1.36, 2.52)	< 0.001	
≥60	461	179	3.00 (2.19, 4.19)	< 0.001	
BMI					0.035
< 24	398	115	2.85 (2.02, 4.12)	< 0.001	
≥24	514	180	1.89 (1.46, 2.48)	< 0.001	
Hypertension					0.73
No	411	70	2.24 (1.59, 3.22)	< 0.001	
Yes	501	225	2.25 (1.73, 2.96)	< 0.001	
HbA1c					0.834
< 7%	372	83	2.34 (1.64, 3.43)	< 0.001	
≥7%	540	212	2.26 (1.74, 2.98)	< 0.001	
Insulin use					0.038
No	589	151	2.90 (2.14, 4.01)	< 0.001	
Yes	323	144	1.86 (1.38, 2.56)	< 0.001	
Diabetic duration					0.731
< 10 years	497	115	2.15 (1.61, 2.92)	< 0.001	
≥10 years	415	180	2.37 (1.75, 3.29)	< 0.001	

ORs (95% CIs) represent the association between each 1-SD increase in UHR and DKD within each subgroup. P for interaction was used to evaluate heterogeneity across subgroups. Estimates were obtained from the fully adjusted model. UHR, uric acid to high-density lipoprotein cholesterol ratio; DKD, diabetic kidney disease; OR, odds ratio; CI, confidence interval; BMI, body mass index; HbA1c, glycated hemoglobin A1c.

Joint analyses of UHR with BMI and insulin use are shown in Table 4. In the UHR–BMI analysis, high UHR was associated with higher odds of DKD in both BMI strata, with adjusted ORs of 3.77 (95% CI, 2.22–6.49) and 3.38 (95% CI, 2.10–5.49) for BMI < 24 and ≥24 kg/m^2^, respectively, whereas BMI ≥24 kg/m^2^ without elevated UHR was not significantly associated with DKD. In the UHR–insulin use analysis, high UHR was associated with higher odds of DKD regardless of insulin use status, with adjusted ORs of 4.64 (95% CI, 2.88–7.61) in non-insulin users and 5.05 (95% CI, 3.00–8.61) in insulin users. Insulin use among participants with low UHR was also associated with increased odds of DKD [OR 2.48 (95% CI, 1.49–4.16)], and the highest DKD prevalence was observed in participants with both high UHR and insulin use.

**Table 4:** Joint effect analyses of UHR with BMI and insulin use for prevalent DKD.

Joint analysis	*N*	Events	Prevalence, %	Adjusted OR (95% CI)	*P*-value
UHR × BMI					
Low UHR + BMI < 24 kg/m^2^	253	49	19.37	Reference	-
High UHR + BMI < 24 kg/m^2^	146	67	45.89	3.77 (2.22, 6.49)	< 0.001
Low UHR + BMI ≥24 kg/m^2^	203	49	24.14	1.18 (0.71, 1.97)	0.527
High UHR + BMI ≥24 kg/m^2^	310	130	41.94	3.38 (2.10, 5.49)	< 0.001
UHR × insulin use					
Low UHR + no insulin use	297	42	14.14	Reference	-
High UHR + no insulin use	292	109	37.33	4.64 (2.88, 7.61)	< 0.001
Low UHR + insulin use	159	56	35.22	2.48 (1.49, 4.16)	< 0.001
High UHR + insulin use	164	88	53.66	5.05 (3.00, 8.61)	< 0.001

The model was adjusted for age, sex, HbA1c, TG, LDL-C, SBP, diabetic duration, hypertension, and insulin use. In the UHR–insulin use joint analysis, the reference group was low UHR with no insulin use, and the model was adjusted for age, sex, HbA1c, BMI, TG, LDL-C, SBP, diabetic duration, and hypertension. UHR was dichotomized according to the median value. DKD, diabetic kidney disease; UHR, uric acid to high-density lipoprotein cholesterol ratio; BMI, body mass index; OR, odds ratio; CI, confidence interval.

### 3.6 Survey-weighted external validation in the NHANES cohort

Baseline characteristics of the NHANES external validation cohort according to UHR quartiles are shown in Supplementary Table S3. Among 2,738 participants with diabetes, 1,067 had DKD. Across increasing UHR quartiles, participants had a lower proportion of females, higher UA and ACR levels, lower HDL-C and eGFR levels, and higher frequencies of insulin use and hypertension. The prevalence of DKD increased progressively from 28.61% in the lowest UHR quartile to 52.49% in the highest quartile (*P* < 0.001).

Survey-weighted logistic regression showed a positive association between UHR and prevalent DKD in the NHANES cohort (Table 5). In the fully adjusted model, each 1-unit increase in UHR was associated with higher odds of DKD (OR, 1.03; 95% CI, 1.02–1.04; *P* < 0.001), and each 1-SD increase was associated with a 44% higher odds of DKD (OR, 1.44; 95% CI, 1.23–1.68; *P* < 0.001). Compared with the low-UHR group, the high-UHR group had higher odds of DKD (OR, 1.81; 95% CI, 1.37–2.40; *P* < 0.001). In quartile analyses, Q4 remained significantly associated with DKD (OR, 2.61; 95% CI, 1.79–3.79; *P* < 0.001), with a significant trend across quartiles (*P* for trend < 0.001).

**Table 5:** Survey-weighted association between UHR and prevalent DKD in the NHANES cohort.

Variables	Model A OR (95% CI)	*P*-value	Model B OR (95% CI)	*P*-value	Model C OR (95% CI)	*P*-value
UHR (per 1 unit)	1.03 (1.02, 1.04)	< 0.001	1.03 (1.02, 1.04)	< 0.001	1.03 (1.02, 1.04)	< 0.001
UHR (per 1-SD)	1.47 (1.28, 1.68)	< 0.001	1.45 (1.25, 1.68)	< 0.001	1.44 (1.23, 1.68)	< 0.001
Median-split						
Low UHR	Reference		Reference		Reference	
High UHR	1.92 (1.47, 2.51)	< 0.001	1.83 (1.38, 2.43)	< 0.001	1.81 (1.37, 2.40)	< 0.001
UHR quartiles						
Q1	Reference		Reference		Reference	
Q2	1.22 (0.85, 1.76)	0.270	1.16 (0.77, 1.74)	0.480	1.16 (0.76, 1.79)	0.487
Q3	1.66 (1.16, 2.38)	0.007	1.54 (1.01, 2.33)	0.043	1.54 (0.99, 2.37)	0.053
Q4	2.76 (2.01, 3.80)	< 0.001	2.62 (1.83, 3.76)	< 0.001	2.61 (1.79, 3.79)	< 0.001
*P* for trend		< 0.001		< 0.001		< 0.001

Model A was adjusted for age, sex, and race/ethnicity. Model B was further adjusted for BMI, SBP, and HbA1c. Model C was further adjusted for diabetic duration, hypertension, and insulin use. Survey-weighted logistic regression was performed using SDMVPSU, SDMVSTRA, and 8-year MEC examination weights. The 8-year MEC weight was calculated as WTMEC2YR divided by 4. UHR median groups and quartiles were defined according to survey-weighted cutoffs. UHR, uric acid to high-density lipoprotein cholesterol ratio; DKD, diabetic kidney disease; OR, odds ratio.

**Figure 4:**
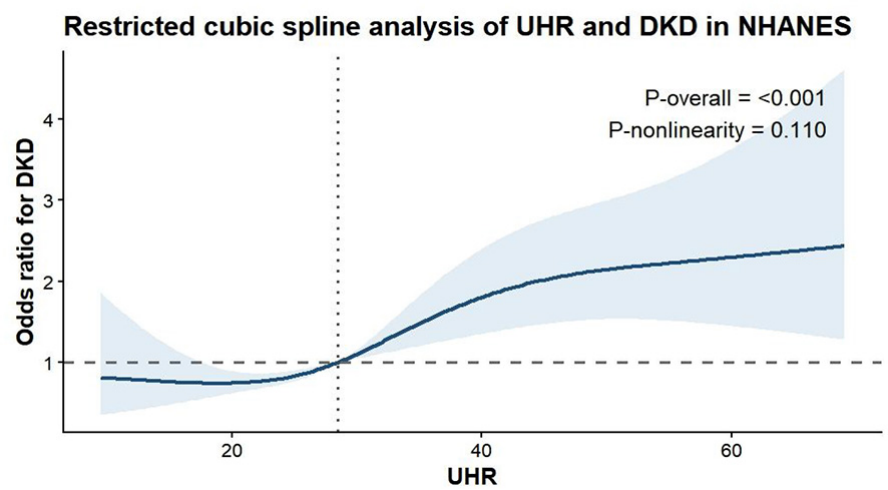
Restricted cubic spline analysis of the association between UHR and prevalent DKD in the NHANES external validation cohort. Restricted cubic spline analysis was performed using survey-weighted logistic regression. The model was fully adjusted for age, sex, race/ethnicity, BMI, SBP, HbA1c, diabetes duration, hypertension, and insulin use. The solid line represents the estimated odds ratio, and the shaded area represents the 95% confidence interval. The horizontal dashed line indicates an odds ratio of 1.0, and the vertical dotted line indicates the weighted median UHR used as the reference value. UHR, uric acid to high-density lipoprotein cholesterol ratio; DKD, diabetic kidney disease;BMI, body mass index; SBP, systolic blood pressure.

Consistent with the logistic regression results, the survey-weighted RCS analysis showed a positive overall association between UHR and DKD in the fully adjusted model (*P*-overall < 0.001), without significant evidence of non-linearity (*P*-non-linearity = 0.110; Figure 4).

In subgroup analyses, the positive association between UHR and DKD was directionally consistent across all examined subgroups (Supplementary Figure S4 and Supplementary Table S4). Per 1-SD increase in UHR, subgroup-specific ORs ranged from 1.33 to 2.25, and none of the interaction terms reached statistical significance.

## 4 Discussion

In this cross-sectional study, higher UHR was consistently associated with greater odds of prevalent DKD among patients with diabetes. This association was observed when UHR was analyzed as a continuous, standardized, median-based, or quartile-based exposure, and it persisted after adjustment for major DKD-related covariates. In the primary cohort, DKD prevalence increased across UHR quartiles, and the highest quartile showed the greatest odds of prevalent DKD. RCS analyses supported a positive overall association between UHR and DKD, although evidence for non-linearity was not significant after full adjustment. Subgroup analyses showed broadly consistent positive associations across clinical strata, with possible effect modification by BMI and insulin use. The positive direction of the association was also observed in the survey-weighted NHANES cohort. The stronger association observed in the primary hospital-based cohort may reflect its tertiary-care setting, where participants likely had a higher burden of metabolic and renal abnormalities than the community-based NHANES sample. Differences in disease severity, sample selection, and residual confounding may also have contributed to the larger effect estimate. Therefore, the difference in OR magnitude between cohorts should be interpreted as cohort heterogeneity rather than inconsistency in the overall association. Taken together, these findings suggest that UHR may reflect an unfavorable renal-metabolic profile associated with DKD, but the cross-sectional design does not allow causal or predictive interpretation.

Our findings are consistent with recent studies suggesting a link between UHR and diabetic kidney involvement. Qin et al., using NHANES 2001–2018 data, reported that higher UHR was associated with greater DKD prevalence among adults with diabetes, with DKD prevalence increasing across UHR quartiles and higher adjusted odds of DKD at higher UHR levels (16). Wei et al. similarly found a positive association in NHANES 2007–2018, although their spline analysis suggested a J-shaped relationship and possible heterogeneity by BMI (21). Beyond type 2 diabetes, a positive association between UHR and prevalent DKD in euthyroid patients with type 1 diabetes was found (22), and more recent work in patients with T2D and NAFLD has extended this question to a metabolically vulnerable subgroup (23). Taken together, these studies support the possibility that UHR is associated with a metabolic profile related to kidney involvement in diabetes.

The novelty of the present study should be viewed as an extension and refinement of existing evidence rather than as the first report of a UHR-DKD association. First, the main analysis was conducted in an independent clinical cohort of patients with T2D, and the association was then examined in a survey-weighted NHANES validation cohort. This two-cohort design provided complementary evidence from both a hospital-based clinical setting and a population-based sample. Second, UHR was evaluated using multiple modeling strategies, including per-unit increase, per-SD increase, median-based categories, and quartiles, and the direction of association remained consistent across these approaches. Third, the RCS analyses supported a positive overall association between UHR and DKD; however, evidence for non-linearity was attenuated after full adjustment in the primary cohort and was not significant in the NHANES validation cohort. This partly differs from the J-shaped pattern reported by Wei et al. (21), and such discrepancy may relate to differences in study cycles, population characteristics, UHR distribution, covariate adjustment, and model specification. Finally, the primary cohort suggested possible heterogeneity by BMI and insulin use, but these interaction findings were not confirmed in the NHANES validation cohort (21, 22). Given the exploratory nature of the subgroup analyses and the absence of formal correction for multiple comparisons, these interaction findings should be interpreted cautiously. Thus, the most reproducible finding across the present and previous studies was the positive direction of the UHR-DKD association, whereas the precise exposure-response shape and potential effect modifiers remain to be clarified.

Beyond the individual roles of uric acid and HDL-C, the present findings may be better understood from the composite nature of UHR as a ratio. A higher UHR reflects the co-occurrence of higher serum uric acid and lower HDL-C levels. Elevated uric acid may contribute to kidney injury through oxidative stress, inflammatory activation, endothelial dysfunction, and altered intrarenal hemodynamics, which may promote glomerular microvascular damage and albuminuria (8, 24–26). Lower HDL-C, in contrast, may indicate weakened antioxidative, anti-inflammatory, and endothelial-protective capacity, particularly in the metabolic context of diabetes (10–12). Therefore, rather than representing a single pathway, UHR may summarize an unfavorable renal-metabolic milieu in which urate-related stress, reduced HDL-related protection, lipid abnormalities, inflammation, oxidative stress, and microvascular dysfunction overlap in patients with T2D. This interpretation is consistent with the baseline pattern in our primary cohort, where higher UHR quartiles were accompanied by progressively higher sCr and ACR levels and lower eGFR, consistent with a more adverse renal profile in participants with higher UHR (Table 1).

Another possible explanation is that UHR may partly reflect broader cardiometabolic dysregulation. Previous studies have linked UHR with insulin resistance and metabolic syndrome (27, 28), both of which are closely related to renal injury in diabetes (24). However, insulin resistance was not directly measured, so this pathway remains hypothesis-generating. In the primary cohort, subgroup and joint analyses suggested possible heterogeneity by BMI and insulin use. These insulin-related findings should be interpreted cautiously, because insulin therapy may indicate longer diabetes duration, poorer glycemic control, advanced metabolic or renal disease, and limited alternative treatment options rather than directly modifying the UHR-DKD association. As these interaction findings were not reproduced in the NHANES validation cohort, the subgroup, interaction, and joint-analysis results should be regarded as exploratory and require confirmation in future studies.

From a clinical perspective, UHR may have exploratory value as a simple composite laboratory indicator in patients with T2D. Both serum uric acid and HDL-C are commonly measured in routine metabolic and lipid assessments, and UHR can be calculated without additional specialized testing when both measurements are available. Compared with either component alone, UHR combines two routinely measured metabolic parameters and may provide a complementary view of the urate–lipid metabolic profile (28). This feature may increase its feasibility in routine care, particularly in settings where more complex biomarkers are not readily available (29).

In the present study, the positive association between UHR and prevalent DKD suggests the potential relevance of this simple composite indicator. DKD prevalence increased from 20.52% in the lowest UHR quartile to 55.26% in the highest quartile in the primary cohort (Table 1), and the positive association persisted after multivariable adjustment and was directionally supported in the NHANES validation cohort (Tables 2, 5). Together with the exposure-response pattern observed in the RCS analyses (Figures 2, 4), these findings suggest that higher UHR may be accompanied by a more adverse renal-metabolic profile in patients with T2D. However, assessment of DKD should continue to rely on established kidney measures such as eGFR and albuminuria (4, 5). Nevertheless, this potential role is adjunctive and exploratory. UHR cannot replace eGFR or albuminuria, nor should it be used alone to diagnose DKD or guide treatment decisions based on the present cross-sectional evidence. Whether UHR provides incremental predictive value beyond established clinical variables remains a question for prospective studies (30, 31). Additionally, because UA is partly determined by renal excretion, impaired kidney function may influence UHR and contribute to the observed association with DKD. To address this concern, we performed additional analyses according to DKD components and repeated the analysis after excluding participants with eGFR < 60 mL/min/1.73 m^2^. The association between UHR and ACR ≥30 mg/g remained significant in participants with preserved eGFR, suggesting that the UHR-DKD association was not solely explained by reduced kidney function. Nevertheless, given the cross-sectional design, reverse causation cannot be excluded.

Several strengths and limitations of this study should be acknowledged. Strengths include the use of two independent cohorts with distinct demographic and clinical profiles, the application of survey-weighted analyses in the NHANES cohort to account for the complex sampling design and improve population representativeness, the use of multiple logistic regression models with sequential covariate adjustment to examine the consistency of the association, the formal evaluation of exposure-response patterns using RCS analyses, as well as subgroup, interaction, and joint analyses to explore heterogeneity across clinically relevant strata. Nevertheless, several limitations warrant caution. First, the cross-sectional design of both cohorts precludes causal inference; in particular, reverse causation is possible because impaired kidney function may increase serum uric acid levels and thereby influence UHR. Although additional analyses by DKD components and analyses excluding participants with eGFR < 60 mL/min/1.73 m^2^ were performed, the residual influence of kidney function on UHR cannot be fully excluded. Therefore, UHR should be interpreted as an index associated with prevalent DKD rather than as a causal, diagnostic, or predictive marker. Second, the primary cohort was derived from a single tertiary care center and consisted of hospitalized patients, which may limit generalizability to community-based populations. Third, DKD classification relied on single-occasion measurements of eGFR and/or ACR, which has substantial within-individual variability; misclassification cannot be excluded. Fourth, although we performed a medication-adjusted sensitivity analysis including SGLT2i/GLP-1RA use and statin use, and adjusted for insulin use in the main models, residual confounding from unmeasured medications and lifestyle factors may remain. In addition, because insulin therapy was not randomly assigned, findings involving insulin-use strata may be influenced by confounding by indication and underlying disease severity. Smoking status was not consistently available in the electronic medical records. Because cigarette smoking may influence HDL-C levels and thereby affect UHR, its potential confounding effect cannot be fully excluded. Information on urate-lowering agents, diuretics, diet, alcohol intake, and physical activity was not consistently available in the electronic medical records and therefore could not be reliably included in the multivariable models. Fifth, the NHANES validation cohort included adults with diabetes and may have included a small proportion of individuals with type 1 diabetes, which may introduce heterogeneity. Finally, whether UHR provides incremental predictive value beyond established clinical variables for incident DKD or its progression remains to be evaluated in prospective longitudinal studies.

## 5 Conclusion

In conclusion, elevated UHR was associated with greater odds of prevalent DKD in a Chinese hospital-based T2D cohort, and the positive direction of this association was also observed in the survey-weighted NHANES validation cohort. These findings suggest that UHR may reflect an unfavorable renal-metabolic profile related to DKD in patients with diabetes. However, given the cross-sectional design and the possibility that kidney dysfunction may itself influence serum uric acid levels, UHR should not be interpreted as a causal factor, diagnostic marker, or predictive tool. Prospective studies with longitudinal kidney outcomes and more detailed medication and lifestyle information are needed to determine whether UHR predicts incident DKD or DKD progression and whether it adds value beyond established kidney measures such as eGFR and albuminuria.

## Data Availability

The data underlying this article will be shared on reasonable request to the corresponding author.
