# Supplementary material for: Cross-sectional association of the uric acid-to-HDL cholesterol ratio with diabetic kidney disease in patients with type 2 diabetes
Source: Front Nutr. 2026 Jul 27;13:1887095. doi: 10.3389/fnut.2026.1887095 (PMC13454203; doi:10.3389/fnut.2026.1887095)
Supplement: Supplementary file 1 [file Data_Sheet_1.docx]

Supplementary Figure S1. Flowchart for selection of the NHANES 2011–2018 analytic sample. HbA1c, glycated hemoglobin A1c; FPG, fasting plasma glucose; UHR, uric acid to high-density lipoprotein cholesterol ratio; eGFR, estimated glomerular filtration rate; ACR, albumin-to-creatinine ratio; DKD, diabetic kidney disease.

Supplementary Figure S2. Assessment of multicollinearity among variables included in the fully adjusted Model 3. (A) Variance inflation factors for variables included in Model 3. UHR, uric acid to high-density lipoprotein cholesterol ratio. (B)Spearman correlation matrix of variables included in Model 3. HbA1c, glycated hemoglobin A1c; BMI, body mass index; TG, triglycerides; LDL, low-density lipoprotein cholesterol; SBP, systolic blood pressure; VIF, variance inflation factor.

**A**

**B**

Supplementary Figure S3. Restricted cubic spline analyses were performed using fully adjusted logistic regression models. Panels show the associations of UHR with ACR ≥30 mg/g, eGFR <60 mL/min/1.73 m^2^, the coexistence of ACR ≥30 mg/g and eGFR <60 mL/min/1.73 m^2^, and ACR ≥30 mg/g after excluding participants with eGFR <60 mL/min/1.73 m^2^. Models were adjusted for age, sex, HbA1c, BMI, TG, LDL-C, SBP, diabetes duration, hypertension, and insulin use. Red solid lines indicate predicted ORs, red dashed lines indicate 95% CIs, and gray histograms indicate the distribution of UHR.

Supplementary Figure S4 Subgroup analysis of the association between UHR and DKD in the NHANES external validation cohort. Odds ratios and 95% confidence intervals for DKD were estimated per 1-standard deviation increase in UHR using the fully adjusted Model C. UHR, uric acid to high-density lipoprotein cholesterol ratio; DKD, diabetic kidney disease;BMI, body mass index.

| Supplementary Table S1. Medication-adjusted sensitivity analysis for UHR and DKD | | |
| --- | --- | --- |
| Variable | Medication-adjusted model OR (95% CI) | P value |
| UHR (per unit) | 1.06 (1.04, 1.08) | <0.001 |
| UHR (per SD) | 2.21 (1.80, 2.75) | <0.001 |
| Median-split |  |  |
| Low UHR | Reference |  |
| High UHR | 3.23 (2.24, 4.69) | <0.001 |
| UHR Quartiles |  |  |
| Q1 | Reference |  |
| Q2 | 1.23 (0.74, 2.05) | 0.429 |
| Q3 | 2.19 (1.31, 3.70) | 0.003 |
| Q4 | 6.45 (3.80, 11.15) | <0.001 |
| P for trend |  | <0.001 |

The medication-adjusted sensitivity model was adjusted for age, sex, HbA1c, BMI, TG, LDL-C, SBP, diabetic duration, hypertension, insulin use, GLP-1 receptor agonist/SGLT2 inhibitor use, and statin use. UHR, uric acid-to-HDL cholesterol ratio; DKD, diabetic kidney disease; OR, odds ratio; CI, confidence interval.

| Supplementary Table S2. Analyses of the association between UHR and DKD components in the primary cohort | | | | | | |
| --- | --- | --- | --- | --- | --- | --- |
| Outcome | Events/N | OR per 1-SD increase in UHR | P value | OR for Q4 vs Q1 | P value | P for trend |
| ACR ≥30 mg/g | 285/912 | 2.05 (1.67, 2.53) | <0.001 | 5.66 (3.34, 9.73) | <0.001 | <0.001 |
| eGFR <60 mL/min/1.73 m² | 68/912 | 2.39 (1.81, 3.25) | <0.001 | 18.10 (6.40, 61.69) | <0.001 | <0.001 |
| ACR ≥30 mg/g and eGFR <60 mL/min/1.73 m² | 58/912 | 2.33 (1.75, 3.19) | <0.001 | 20.88 (6.66, 83.11) | <0.001 | <0.001 |
| ACR ≥30 mg/g | 227/844 | 1.93 (1.55, 2.42) | <0.001 | 4.39 (2.51, 7.80) | <0.001 | <0.001 |

ORs were estimated using logistic regression models. UHR was analyzed per 1-SD increase and by quartiles, with Q1 as the reference group. All the models were adjusted for age, sex, HbA1c, BMI, TG, LDL-C, SBP, diabetes duration, hypertension, and insulin use. The analysis in the last row was performed after excluding participants with eGFR <60 mL/min/1.73 m². Analyses involving reduced eGFR should be interpreted cautiously because of the limited number of events. UHR, uric acid to high-density lipoprotein cholesterol ratio; UACR, urinary albumin-to-creatinine ratio; eGFR, estimated glomerular filtration rate; DKD, diabetic kidney disease; OR, odds ratio.

| Supplementary Table S3. Baseline characteristics of the NHANES external validation cohort | | | | | |
| --- | --- | --- | --- | --- | --- |
| Variable | Q1 (n=685) | Q2 (n=685) | Q3 (n=684) | Q4 (n=684) | P value |
| Age, years | 60.79±13.00 | 61.28±12.96 | 61.92±12.29 | 62.34±13.15 | 0.121 |
| Female, n(%) | 456 (66.57) | 359 (52.41) | 260 (38.01) | 200 (29.24) | <0.001 |
| Race/ethnicity, n(%) |  |  |  |  | <0.001 |
| Non-Hispanic Black | 191 (27.88) | 200 (29.20) | 160 (23.39) | 150 (21.93) | <0.001 |
| Non-Hispanic White | 167 (24.38) | 201 (29.34) | 234 (34.21) | 274 (40.06) |  |
| Mexican American | 140 (20.44) | 116 (16.93) | 118 (17.25) | 97 (14.18) |  |
| Other Hispanic | 95 (13.87) | 73 (10.66) | 61 (8.92) | 67 (9.80) |  |
| Other Race - Including Multi-Racial | 92 (13.43) | 95 (13.87) | 111 (16.23) | 96 (14.04) |  |
| BMI, kg/m² | 30.24±6.86 | 32.06±7.61 | 32.89±7.39 | 34.30±7.73 | <0.001 |
| SBP, mmHg | 132.73±19.82 | 132.06±20.18 | 132.81±18.79 | 130.74±20.05 | 0.198 |
| DBP, mmHg | 69.56±13.29 | 68.99±15.19 | 69.54±13.25 | 67.33±15.18 | 0.013 |
| HbA1c, % | 7.52±2.04 | 7.57±1.95 | 7.43±1.64 | 7.50±1.59 | 0.528 |
| HDL-C, mmol/L | 1.60±0.39 | 1.30±0.25 | 1.12±0.19 | 0.93±0.18 | <0.001 |
| UA, μmol/L | 249.82 (214.13, 291.45) | 309.30 (273.61, 350.93) | 350.93 (315.24, 392.57) | 428.26 (374.72, 481.79) | <0.001 |
| UHR | 16.87 (14.02, 19.17) | 24.41 (22.57, 26.03) | 31.70 (29.73, 34.06) | 44.74 (39.95, 52.08) | <0.001 |
| ACR, mg/g | 11.83 (7.07, 30.00) | 13.51 (7.01, 44.35) | 14.16 (7.17, 49.81) | 17.36 (7.64, 105.52) | <0.001 |
| eGFR, mL/min/1.73 m² | 100.90 (89.03, 110.96) | 96.49 (77.80, 107.56) | 90.50 (68.57, 103.22) | 76.33 (55.09, 98.76) | <0.001 |
| Diabetic duration, years | 10.00 (5.00, 18.00) | 10.00 (4.00, 18.00) | 10.00 (4.00, 17.00) | 10.00 (3.00, 17.00) | 0.336 |
| Insulin use, n(%) | 189 (27.59) | 178 (25.99) | 177 (25.88) | 249 (36.46) | <0.001 |
| Hypertension, n(%) | 478 (69.78) | 500 (73.31) | 511 (74.71) | 549 (80.26) | <0.001 |
| DKD prevalence, n (%) | 196 (28.61) | 240 (35.04) | 272 (39.77) | 359 (52.49) | <0.001 |

BMI, body mass index; SBP, systolic blood pressure; DBP, diastolic blood pressure; HbA1c, glycated hemoglobin A1c; HDL-C, high-density lipoprotein cholesterol; UA, uric acid; UHR, uric acid to high-density lipoprotein cholesterol ratio; UACR, urinary albumin-to-creatinine ratio; eGFR, estimated glomerular filtration rate; DKD, diabetic kidney disease.

| Supplementary Table S4. Survey-weighted subgroup and interaction analyses of the association between UHR and DKD in the NHANES external validation cohort | | | |
| --- | --- | --- | --- |
| Subgroup | OR (95%CI) | P value | P for interaction |
| Sex |  |  | 0.554 |
| Female | 1.34 (1.00, 1.79) | 0.047 |  |
| Male | 1.48 (1.22, 1.81) | <0.001 |  |
| Age |  |  | 0.628 |
| <60 | 1.39 (1.08, 1.80) | 0.011 |  |
| ≥60 | 1.48 (1.23, 1.77) | <0.001 |  |
| BMI |  |  | 0.331 |
| <24 | 2.25 (1.47, 3.44) | <0.001 |  |
| ≥24 | 1.44 (1.25, 1.67) | <0.001 |  |
| Hypertension |  |  | 0.185 |
| No | 1.75 (1.23, 2.48) | 0.002 |  |
| Yes | 1.38 (1.17, 1.63) | <0.001 |  |
| HbA1c |  |  | 0.815 |
| <7% | 1.33 (1.05, 1.68) | 0.018 |  |
| ≥7% | 1.56 (1.35, 1.81) | <0.001 |  |
| Insulin use |  |  | 0.309 |
| No | 1.35 (1.09, 1.66) | 0.005 |  |
| Yes | 1.65 (1.34, 2.05) | <0.001 |  |
| Diabetic duration |  |  | 0.78 |
| <10 years | 1.43 (1.20, 1.70) | <0.001 |  |
| ≥10 years | 1.43 (1.16, 1.75) | <0.001 |  |

ORs and 95% CIs represent the association between each 1-SD increase in UHR and DKD within each subgroup. Estimates were obtained from the fully adjusted for age, sex, race/ethnicity, BMI, SBP, HbA1c, diabetic duration, hypertension, and insulin use, except for the stratification variable where appropriate. Survey-weighted logistic regression was performed using SDMVPSU, SDMVSTRA, and 8-year MEC examination weights. UHR, uric acid to high-density lipoprotein cholesterol ratio; DKD, diabetic kidney disease; OR, odds ratio; CI, confidence interval; BMI, body mass index; HbA1c, glycated hemoglobin A1c.
